# Dysregulated miRNAs Targeting Adiponectin Signaling in Colorectal Cancer

**DOI:** 10.3390/ijms26157196

**Published:** 2025-07-25

**Authors:** Momchil Barbolov, Svetla Slavova, Neda Nedeva, Krasimir Ivanov, Nikola Kolev, Katarzyna Komosinska-Vassev, Diana Ivanova, Deyana Vankova, Yoana Kiselova-Kaneva

**Affiliations:** 1 Department of Biochemistry, Molecular Medicine and Nutrigenomics, Faculty of Pharmacy, Medical University “Prof. Dr. Paraskev Stoyanov”, 9000 Varna, Bulgaria; momchil.barbolov@mu-varna.bg (M.B.); neda.nedeva@mu-varna.bg (N.N.); divanova@mu-varna.bg (D.I.); deyana.vankova@mu-varna.bg (D.V.); 2 Department of Biology, Faculty of Pharmacy, Medical University “Prof. Dr. Paraskev Stoyanov”, 9000 Varna, Bulgaria; svetla.slavova@mu-varna.bg; 3 Department of General and Operative Surgery, Faculty of Medicine, Medical University “Prof. Dr. Paraskev Stoyanov”, 9002 Varna, Bulgaria; kdivanov@abv.bg (K.I.); nikola.kolev@mu-varna.bg (N.K.); 4 Department of Clinical Chemistry and Laboratory Diagnostics, Faculty of Pharmaceutical Sciences in Sosnowiec, Medical University of Silesia in Katowice, 40-055 Katowice, Poland; kvassev@sum.edu.pl

**Keywords:** colorectal cancer, adiponectin signaling, miRNAs

## Abstract

Dysregulation in miRNA expression has been reported in a variety of tumors, including colorectal cancer (CRC), where adiponectin regulates a number of processes related to tumorigenesis. The aim of this study was to identify a panel of heavily and consistently altered miRNAs in CRC that affect adiponectin signaling based on bioinformatics analysis and cross-referencing the available literature. Bioinformatics tools were used to analyze publicly available datasets to identify miRNAs targeting the adiponectin pathway that are substantially dysregulated in CRC. In parallel, a comprehensive literature review was conducted to gather and explore existing knowledge on the relationship between CRC, adiponectin signaling, and miRNA dysregulation. Bioinformatics analysis revealed a set of miRNAs that target adiponectin signaling and are consistently altered in CRC. Several candidate miRNAs, including miR-215-5p, miR-340-5p, miR-181a-5p, miR-150-5p, miR-96-5p, miR-19a-3p, and miR-21-5p, were identified as potential key regulators of the adiponectin cascade, while also being systemically dysregulated in CRC. Through gene ontology enrichment analysis, we further elucidated the biological processes and pathways impacted by these miRNAs, providing insight into their contributions to CRC. The literature review did not identify any previously reported shared connection between these miRNAs, adiponectin signaling, and CRC pathogenesis.

## 1. Introduction

MicroRNAs are short, non-coding nucleic acids, typically 20–24 nucleotides long, that play a crucial role in post-transcriptional gene modulation, most commonly through downregulation [1]. Multiple studies have shown dysregulation of miRNA expression in various human malignancies such as colon [2], oral [3], breast [4], cervical [5], lung [6], and pancreatic cancers [7], and in chronic lymphocytic leukemia [8]. Over the past twenty years, the roles of various miRNAs in the pathogenesis of colorectal cancer (CRC) using both in vitro and in vivo models have been investigated [9,10]. For example, as early as 2003, decreased levels of miR-143 and miR-145 in colorectal malignancy were reported [11]—the first instance of proof for a tumor-suppressive effect of miRNAs.

CRC is a malignant disease presenting an increasing incidence globally, which makes it a significant public healthcare and socio-economical challenge [12]. Well-known risk factors such as unhealthy dietary habits, poor physical activity, and excessive body fat are associated with over 50% of CRC cases [13]. Colorectal cancer comprises a group of heterogeneous malignancies differing by microsatellite instability, chromosomal instability, and CpG island methylations, each with distinct genetic and epigenetic alterations, which impact tumor behavior and therapy response [14]. Additionally, defined by the previously mentioned characteristics, as well as other features like metabolic and signaling pathway alterations, the transcriptome-based consensus molecular subtypes (CMS1-4) classification has been proposed by the CRC Subtyping Consortium [15]. The strong relation between obesity and CRC has been described in many epidemiological studies over the past 30 years [16,17]. The mechanisms by which obesity promotes CRC are multifactorial and not fully elucidated yet, but known to involve chronic inflammation, insulin resistance, neoangiogenesis, and alterations in adipokine levels [18,19].

**Figure 1:**
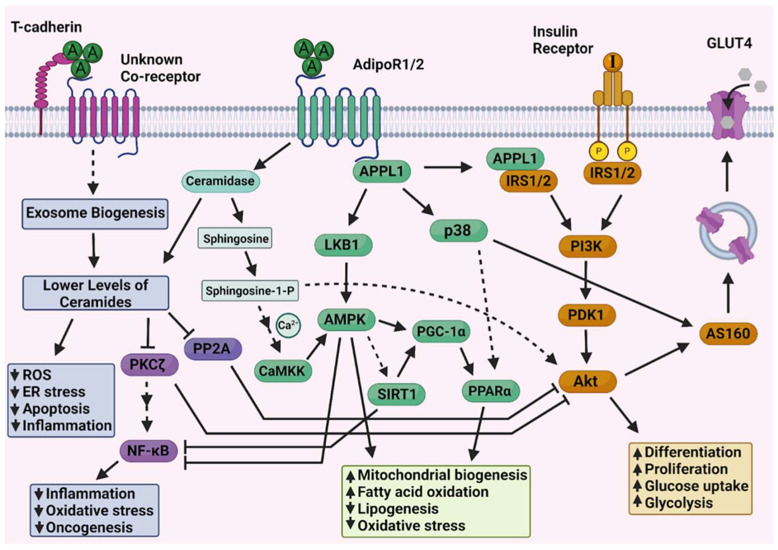
Adiponectin signaling cascade and its relation to insulin signaling. Insulin-sensitizing effects of adiponectin. Solid lines represent direct interactions (arrows for activation and blunt-end lines for inhibition), whereas dashed lines represent indirect activation. Figure generated with BioRender https://www.biorender.com/. T-cadherin, Tissue Cadherin; AdipoR1/2, Adiponectin Receptor 1/2; IRS1/2, Insulin Receptor Substrate 1/2; PI3K, Phosphoinositide 3-Kinase; PDK1, Phosphoinositide-Dependent Kinase 1; Akt, Protein Kinase B; GLUT4, Glucose Transporter Type 4; AS160, Akt Substrate of 160 kDa;APPL1, Adaptor Protein, Phosphotyrosine Interacting with PH Domain and Leucine Zipper 1; LKB1,Liver Kinase B1; AMPK, AMP-Activated Protein Kinase; CaMKK, Calcium/Calmodulin-Dependent Protein Kinase Kinase; p38, p38 Mitogen-Activated Protein Kinase;SIRT1, Sirtuin 1; PGC-1α, Peroxisome Proliferator-Activated Receptor Gamma Coactivator 1-Alpha; PPARα,Peroxisome Proliferator-Activated Receptor Alpha; PP2A, Protein Phosphatase 2A; PKCζ, Protein Kinase C Zeta; NF-κB, Nuclear Factor Kappa B; ROS, Reactive Oxygen Species; ER, Endoplasmic Reticulum; Ca^2+^, Calcium Ion; Sphingosine-1-P, Sphingosine-1-Phosphate.

One of these adipokines, adiponectin, possesses strong anti-tumorigenic effects [20]. Typically reduced in obesity, it exerts anti-inflammatory, anti-proliferative, and insulin-sensitizing effects on a variety of cell types [21]. Engaging the adiponectin receptors, ADIPOR1, ADIPOR2, and T-cadherin, it activates signaling cascades such as the AMP-activated protein kinase (AMPK) and the peroxisome proliferator-activated receptor alpha (PPARα) pathways, thus enhancing insulin sensitivity, promoting fatty acid oxidation, and suppressing inflammation [21]. Adiponectin signaling and its interplay with insulin cascades is depicted in Figure 1. T-cadherin is a glycosylphosphatidylinositol (GPI)-anchored protein with no cytosolic domains, meaning that it cannot transduce signals from the surface by itself, yet AdipoR1/2-independent effects have been described for T-cadherin [22], hinting at the presence of a still unknown adiponectin co-receptor. T-cadherin and adiponectin are endocytosed upon ligation with the co-receptor and sorted into multi-vesicular bodies and recycling endosomes, which, via a yet unclear mechanism, are additionally loaded exclusively with ceramides before being exocytosed back to the surface, thus significantly lowering intracellular ceramide levels [23]. Meanwhile, engagement of AdipoR1/2 leads to the activation of their cytosolic ceramidase domains [24] further decreasing intracellular ceramides. The combined effect of ceramide depletion enhances insulin signaling, as ceramides are direct activators of the non-canonical PKCζ and the PP2A phosphatase [25], both of which normally deactivate the Akt kinase, central to insulin signaling. Lack of ceramides also improves the redox status and relieves ER stress in the cell [26]. Additionally, PKCζ is a powerful activator of the NF-κB pro-inflammatory pathway, which is also downregulated by low ceramide levels [27]. The major metabolic effect of adiponectin signaling is the activation of AMPK, central for energy utilization from lipid catabolism. AMPK is activated by two axes—direct recruitment of LKB1 by the APPL1 adaptor of AdipoR1/2, as well as CaMKKβ-mediated phosphorylation, initiated by sphingosine-1-phosphate [28]. Sphingosine is generated by the ceramidase activity of AdipoR1/2 and further phosphorylated to S-1-P, which signals in an autocrine manner via GPCRs on the surface to initiate calcium influx [29]. AMPK activity results in downregulation of lipid synthesis and upregulation of fatty acid oxidation, mitochondrial biogenesis, and ultimately improved redox and energy status via PGC-1α-mediated activation of the PPARα transcription factor [30]. SIRT1, a deacetylase carrying out many of the positive effects of caloric restriction, contributes indirectly to PPARα activity, while also neutralizing NF-κB signaling [31]. The APPL1 adaptor is also central to the insulin-sensitizing effects of adiponectin, as it can directly interact with the IRS1/2 [32], initiating the downstream effects of Akt activity such as improved cellular function and glucose metabolism. Adiponectin can increase GLUT4 translocation and subsequent glucose uptake in insulin-dependent tissues either by Akt-mediated or p38-mediated inactivation of AS160, which allows for vesicle efflux. Additionally, p38 activity leads to improved transcription of PPARα, further enhancing its effects. In summary, adiponectin improves insulin-sensitivity by lowering ceramide levels and contributing to the activation of downstream insulin signaling. This results in improved metabolism of glucose and lipids, as well as decreased basal inflammation—protective factors against oncogenesis.

In this study, we employed data mining techniques to select differentially expressed miRNAs in CRC, known to target the above pathways, for future experimental utilization. Understanding the intricate interplay between miRNAs and adipokine signaling will extend our understanding of the mechanisms of CRC development and elucidate new biomarkers for managing obesity-related colorectal cancer.

Our goal was to identify and select a panel of dysregulated miRNAs in CRC that affect adiponectin signaling based on an integrated approach that includes bioinformatics analysis and cross-referencing with a review of the literature. Through gene ontology (GO) enrichment analysis, we further aimed to explore the biological processes and pathways impacted by these miRNAs, providing insight into their contributions to CRC malignancy. The role of the selected miRNAs in CRC pathogenesis and progression would be further validated in future research.

## 2. Results

By selecting key proteins in the adiponectin cascade (Figure 1), we identified which of the significantly dysregulated miRNAs target the aforementioned proteins’ mRNA transcripts, by scraping the miRTarBase database. Then, we selected 7 miRNAs, out of the starting 292, which are both vastly altered across multiple datasets and target the upstream, unique components of the adiponectin pathway—T-cadherin, AdipoR1/2, or APPL1—and separated them into upregulated (3) and downregulated (4) groups.

### 2.1. Database Cross-Referencing

**Figure 2:**
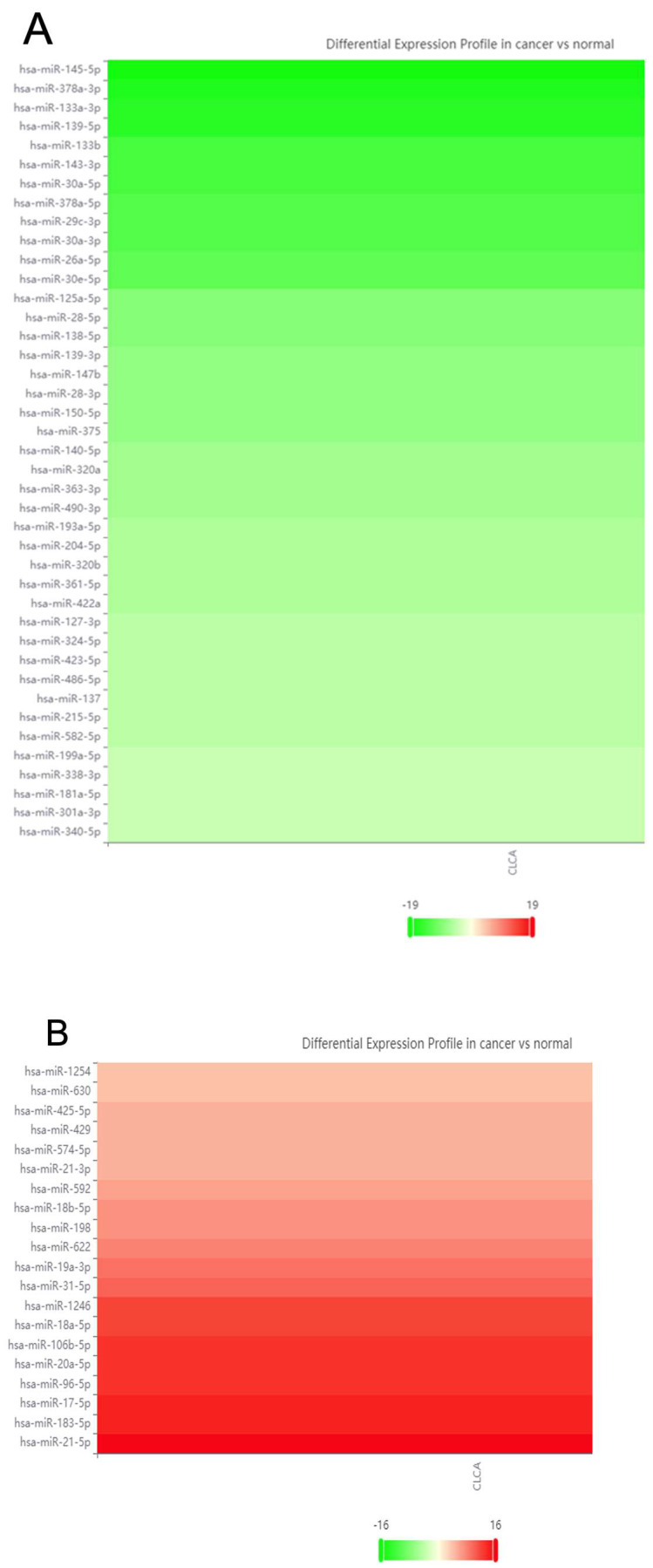
Meta-profiling heatmaps of most consistently dysregulated miRNAs in colorectal cancer tissue vs. normal tissue. (**A**) Downregulated miRNAs in colorectal cancer tissue vs. normal tissue. The green gradient represents the VC score decrease of miRNAs across multiple cancer vs. normal datasets, available from the dbDEMC 3.0 database from −19 (darkest green) to −4 (lightest green). Decrease of these miRNAs in CRC potentially dubs them tumor-suppressive, while some of the genes they regulate would potentially be oncogenes. (**B**) Upregulated miRNAs in colorectal cancer tissue vs. normal tissue. The red gradient represents the VC score increase of miRNAs across multiple cancer vs. normal datasets, available from the dbDEMC 3.0 database from +4 (lightest red) to +16 (darkest red). Increase of these miRNAs in CRC potentially means they could be oncogenic, while some of the genes they regulate would potentially be tumor-suppressors. Images were generated via the meta-profiling feature of dbDEMC 3.0.

Our data mining workflow commenced with the identification of all documented dysregulated miRNAs in colorectal cancer tissues, according to the miRCancer database. Of the 612 entries for which there was referenced experimental evidence, there were 292 individual miRNAs listed. For some there were conflicting reports, stating that they are either downregulated or upregulated, depending on the study. miRNAs were cross-referenced with the dbDEMC 3.0 database, using the meta-profiling heatmap feature. This step shows the average fold-change for the miRNAs between healthy and colorectal cancer tissues, evaluated across multiple datasets, and represented as VC scores. Based on their VC scores, only miRNAs with at least 4-fold average change and above were selected for further analysis—62 significantly, consistently dysregulated miRNAs (Figure 2).

Meanwhile, based on the literature search regarding adiponectin signaling, 16 genes were selected as representative for the pathway—adiponectin itself, AdipoR1&2, their adaptor APPL1, T-cadherin, p38 MAPK, PKCζ, AMPK subunits, PPARα, and the insulin pathway components IRS1&2, Akt, and GSK3β. Using the miRTarBase miRNA target repository, 28 of the 62 previously selected miRNAs were found to target one or more of the chosen genes’ mRNA transcripts. In order to reduce the complexity of the following analysis, from here we focused only on upstream, unique components of the adiponectin pathway—AdipoR1, AdipoR2, T-cadherin, and APPL1, as the insulin pathway exhibits cross-talk with multiple other cell signaling cascades. Through subsequent miRTarBase queries, we found 7 of the 28 selected miRNAs that target those 4 genes—4 miRNAs that are significantly downregulated in colorectal cancer—miR-215, miR-340, miR-181a, and miR-150; and 3 miRNAs that are significantly upregulated in colorectal cancer—miR-96, miR-19a, and miR-21 (Table 1).

These miRNAs are both consistently and substantially dysregulated in CRC and target upstream components of the adiponectin cascade, which suggests they impact oncogenesis (upregulated miRNAs) and tumor suppression (downregulated miRNAs) by virtue of adiponectin signaling regulation, placing them as viable candidates for future experimental work and potential biomarkers for disease management. Of note is that the three chosen upregulated miRNAs each target at least three genes from the adiponectin cascade and, in CRC tissues, their corresponding protein expression, and therefore adiponectin signaling, would be noticeably reduced, while downregulated miRNAs only target one to two genes from the pathway. Considerations regarding the individual miRNAs are expanded on in the Section 3.

### 2.2. Ontology Networks

**Figure 3:**
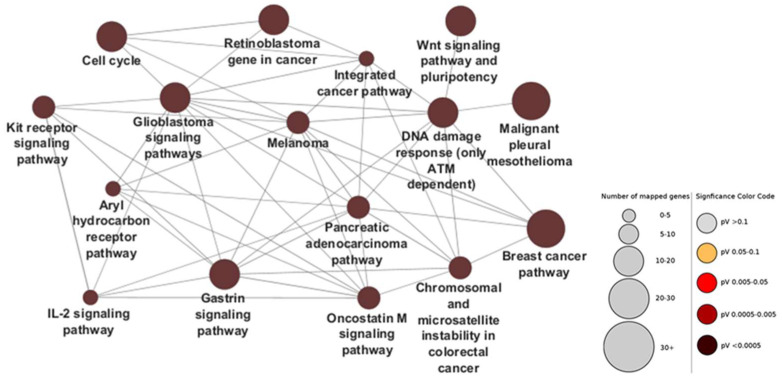
Gene ontology network for all known targets of the most downregulated in CRC, adiponectin pathway-targeting miRNAs. Using miRTarBase, 1587 genes were found to be targeted by the four selected downregulated miRNAs, meaning that the expression of those genes and the activity of their associated pathways should be upregulated in CRC tissues. Circle sizes indicate number of mapped genes to pathway, while color indicates *p* values. Generated via Cytoscape’s ClueGO application, using the WikiPathways database.

**Figure 4:**
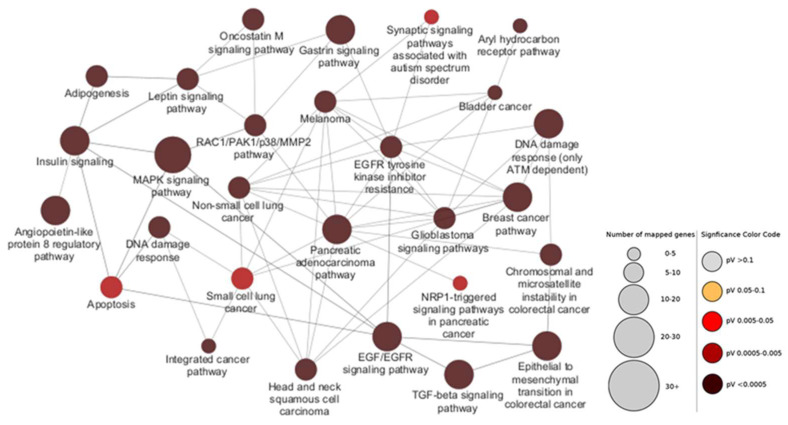
Gene ontology network for all targets of the most upregulated in CRC, adiponectin pathway-targeting miRNAs. Using miRTarBase, 1329 genes were found to be targeted by the three selected upregulated miRNAs, meaning that the expression of those genes and the activity of their associated pathways should be downregulated in CRC tissues. Circle sizes indicate number of mapped genes to pathway, while color indicates *p* values. Generated via Cytoscape’s ClueGO application, using the WikiPathways database.

In order to evaluate which other pathways are affected by the selected seven miRNAs, we constructed gene ontology networks for all genes impacted by the four downregulated and three upregulated miRNAs. For that purpose, we employed the ClueGo application in CytoScape, using the WikiPathways database (see Methods). The miRNA-targeted genes were inferred by consulting the miRTarBase repository. A total of 1587 individual targets were found for the four downregulated (Figure 3) and 1329 targets for the three upregulated miRNAs (Figure 4).

As expected, a large percentage of the genes targeted by selected miRNAs dysregulated in CRC are involved in several other types of cancer as well—retinoblastoma, pleural mesothelioma, breast cancer, pancreatic adenocarcinoma, and so forth. Hypothetically, if the miRNAs targeting those genes are downregulated, the expression of the genes themselves should rise. Besides the concrete oncogenic pathways, other notable nodes in the network include the c-Kit signaling pathway, oncostatin M signaling, the ATM-dependent DNA damage response, the IL-2 pathway, and gastrin signaling, the activities/responsiveness of which should all be increased owing to the downregulation of the miRNAs that target them.

Unsurprisingly, the genes controlled by the dysregulated miRNAs are heavily involved in the progression of various cancers, with a large degree of overlap with the nodes of the previous figure (Supplementary Figure S2). Some notable pathways in which these genes participate are insulin/leptin signaling, adipogenesis, apoptosis cascades, and growth factor (EGF, TGF-β) and MAPK pathways, with the largest amount of submitted genes assigned to the latter. All of those networks should, hypothetically, exhibit lower activity due to the increase of the miRNAs that target them.

### 2.3. MirNET—Most Targeted Genes

**Figure 5:**
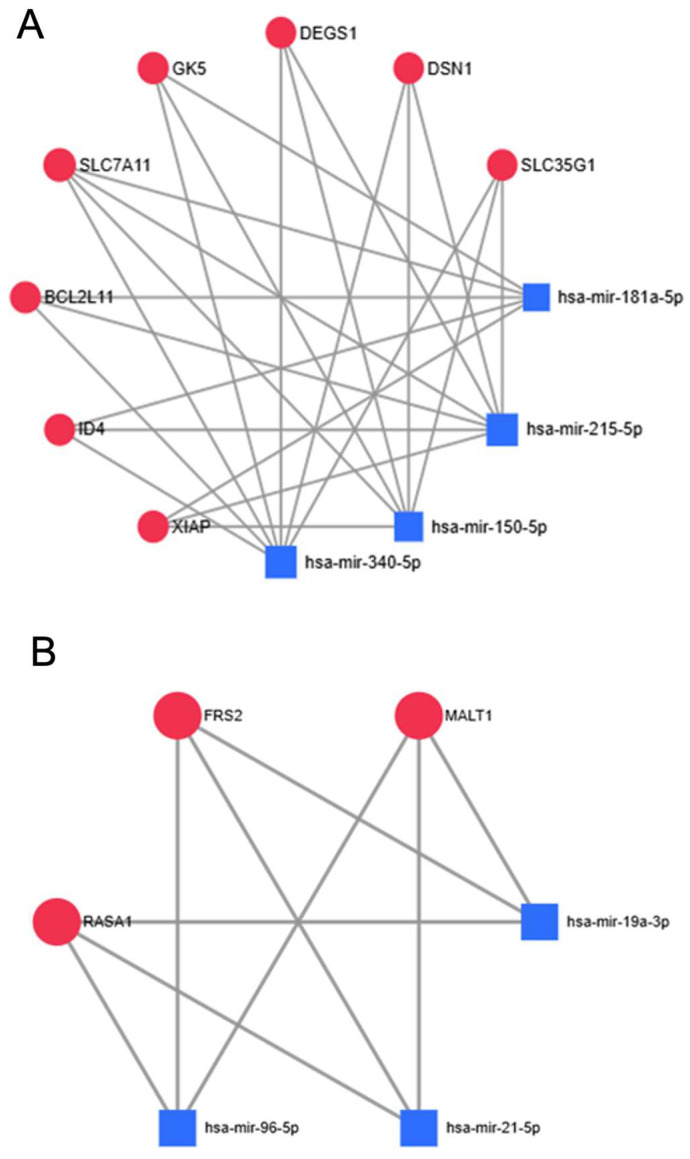
Simplified networks, depicting the most-targeted genes by the selected dysregulated miRNAs. (**A**) Downregulated miRNAs—these genes’ mRNA transcripts are targeted by at least three of the four miRNAs, meaning that their expression should be among the most upregulated in CRC tissues, i.e., they are potential oncogenes or prognostic markers. (**B**) Upregulated miRNAs—these genes’ mRNA transcripts are targeted by all three miRNAs; thus, their expression should be among the most downregulated in CRC tissues, i.e., they are potential tumor-suppressors or prognostic markers. Image generated through the network builder feature of MirNET analytics platform, degree cutoff value set at 2.

Finally, to assess which genes are impacted by multiple of the adiponectin pathway-regulating altered miRNAs, and would, therefore, be likely severely dysregulated themselves, we utilized the network building analytics platform MirNET. For the four downregulated miRNAs, eight genes were found to be targeted by at least three of them—SCL35G1, DSN1, DEGS1, GK5, SLC7A11, XIAP, BCL2L11, and ID4, while for the three upregulated miRNAs, three genes were found to be targeted by all three miRNAs—MALT1, FRS2, and RASA1 (Figure 5).

The genes presented on Figure 5A targeted by selected downregulated miRNAs are expected to be more expressed in colorectal cancer tissues. Their known characteristics are reported in Supplementary Table S1 [33,34,35,36,37,38,39,40,41,42,43,44,45,46,47,48,49,50]. The most heavily targeted genes by our selected upregulated miRNAs, shown on Figure 5B, should in theory be downregulated in colorectal cancer. Their features are summarized in Supplementary Table S2 [51,52,53,54,55]. Briefly, the results of our data mining efforts underscore the possibility of picking out individual miRNAs and target genes from the sea of complexity that is the human transcriptome for future experimental validation through a relatively simplistic workflow protocol, based on available data. The miRNAs and target genes selected through this method do in fact have known connections to metabolism dysregulation and cancer progression.

### 2.4. Literature Review of Reproducibly Altered miRNAs in CRC

To highlight the link between miRNA dysregulation in CRC tissues and their roles in initiation, progression, and prognosis, we summarized findings from reviewing the available literature in Supplementary Tables S3–S6. Supplementary Table S3 [56,57,58,59,60,61,62,63,64,65,66,67,68,69,70,71,72,73,74,75,76,77,78,79,80,81,82,83] lists miRNAs with pro-tumorigenic roles in CRC patients, influencing stages from initiation to metastasis. These oncogenic miRNAs are highly expressed in CRC and promote cell growth, proliferation, invasion, and metastasis. In contrast, anti-tumorigenic miRNAs that are significantly lowered in CRC patient tissues inhibit CRC cell growth, migration, invasion, and induce apoptosis via specific pathways—presented in Supplementary Table S4 [84,85,86,87,88,89,90,91,92,93,94,95,96,97,98,99,100]. In addition, Supplementary Table S5 [61,62,63,64,65,66,67,68,69,70,71,72,80,101,102,103,104,105] and Table S6 [85,87,90,92,93,94,95,96,98,106,107,108,109,110] detail data from in vitro studies of known miRNAs altered in CRC. Respectively, oncogenic microRNAs that promote CRC progression are listed in Table S5, and miRNAs that suppress cancer progression are reported in Table S6.

## 3. Discussion

Based on the bioinformatics analysis performed, we selected a panel of seven miRNAs (four downregulated and three upregulated) that both have consistently dysregulated expression in CRC tissue and cell lines and target key components of the adiponectin signaling cascade. A subsequent literature review of articles from the last twenty years corroborated our findings, illustrating that the cross-utilization of bioinformatics tools with data analysis from the comprehensive review could be a useful method to design a panel of certain microRNAs specific to this type of malignancy. There is ample evidence in literature of the involvement of the miRNAs selected by computational methods in colorectal cancer progression and severity, cementing the notion that potentially relevant miRNAs and their targets can be uncovered by data mining alone.

The present study provides novel insights on the critical role of dysregulated microRNAs in colorectal cancer and their influence on adiponectin signaling pathways. Our data mining approach focused on identifying miRNAs that are known to be dysregulated in CRC and also known to target genes involved in adiponectin signaling, such as ADIPOR1, ADIPOR2, APPL1, and AMPK subunit genes, without relying on prior knowledge of evidence regarding the role of these miRNAs in CRC progression. The adiponectin signaling disruption, driven by these miRNAs, could be key for the pathogenesis of CRC in the context of obesity.

Several miRNAs: miR-150, miR-340, miR-215, and miR-181a from the downregulated ones; and miR-96, miR-21, and miR-19 from the upregulated ones, emerged as promising candidates for key regulators, exhibiting consistently altered expression levels in CRC tissues compared to healthy controls, as inferred through miRCancer and dbDEMC 3.0 cross-referencing. Furthermore, gene ontology (GO) enrichment analysis highlighted how these miRNAs regulate key metabolic and oncogenic pathways, affecting cellular proliferation, differentiation, and apoptosis. In addition, our results are in line with the largest-to date European cross-cancer genome wide association (GWAS) study that estimated the genetic correlation between six solid cancers [111]. Some known evidence about the selected miRNAs with regards to CRC are presented here.

**miR-150** has been shown to be decreased in CRC cell lines SW480 and HT-29, which promotes cancer progression through an enhancing effect on the Wnt pathway, while, conversely, its overexpression restrains the expression of β-catenin, c-myc and CyclinD1 [112]. MiR-150 also suppresses VEGFA/VEGFR2 and downstream Akt signaling in cells from CRC tissues and inhibits cell proliferation, migration, and angiogenesis both in vitro and in vivo [97]. It also downregulates the oncogene iASPP (p53 inhibitor) in SW480 and HCT116 CRC cell lines, resulting in markedly decreased proliferation and invasion [113].

**miR-340** is involved in tumor suppression in CRC by modulating alternative splicing of the PKM gene [114] or specifically targeting RLIP76 [115]. Notably, miR-340 has been proven to suppress proliferation and induce apoptosis of colon cancer cells by regulating an important oncogene, REV3L [116]. In addition, it has been demonstrated that miR-340 is significantly downregulated in bone marrow EpCAM(+) cells of CRC patients with liver metastasis, suggesting that miR-340 could have an essential function in metastatic CRC [117].

**miR-215** expression has been linked to protective effects in several types of cancer, including CRC. Low expression of miR-215-5p has been associated with decreased CRC patient survivability [118], while its overexpression has been proven to decrease tumor volume and invasiveness in vitro and liver metastases in vivo [119]. Additionally, this miRNA targets the EGFR pathway by downregulating the transcription factor HOXB9, which normally promotes the expression of the EGFR ligand epiregulin, thus decreasing CRC cell proliferation [96]. MiR-215 can also affect inflammation and the epithelial-mesenchymal transition by downregulating TRAF5 and IκB-α 40 and FOXM1 in vitro and in vivo [120].

**miR-181a**. It has been reported that miR-181a-5p inhibits cancer cell migration and angiogenesis by downregulating matrix metalloproteinase-14 expression [100]. More evidence of miR-181’s tumor suppressive role comes from studies on long non-coding RNAs. The oncogenic lncRNA CRNDE has been shown to downregulate miR-181a-5p, which in turn causes enhancement of Wnt/β-catenin signaling, while miR-181-5p overexpression caused decreased proliferation and chemoresistance in HCT116 and SW480 cell lines [121]. However, another study has reported that CRC cells with high metastatic potential released more extracellular vesicles enriched in miR-181a-5p compared to cells with low metastatic potential [122]. This dual role of miR-181 in colorectal cancer may stem from stage-specific expression or its decrease due to increased exosome-mediated export.

**miR-96** has been positively associated with increased metastasis and chemoresistance in several cancer types. Its downregulation has been shown to result in the inhibition of tumor growth in mouse CRC models, which occurs through upregulation of the PRKAA2 gene—an AMPK subunit [123]. Knockdown of this miRNA results in increased sensitivity to the anticancer drug oxaliplatin [124]. Several genes involved in the negative regulation of cell migration are also under the inhibitory control of miR-96, which would explain why its overexpression leads to increased cancer invasiveness [125].

**miR-19a** also has stimulatory effects on CRC cell proliferation and migration—it causes lower expression of the pro-apoptotic TIA1 [126], the chloride channel accessory CLCA4 [127], and the FOXF2 transcription factor [104], the latter two being potent inhibitors of the Wnt pathway and thus their decreased expression enhances proliferation and invasion. Furthermore, miR-19a inhibits ferroptosis in HT29 colorectal cancer cells by downregulating the iron sensor IREB2, resulting in less iron accumulation and therefore lower chance of ferroptosis occurring [128].

**miR-21** is one of the most researched oncogenic miRNAs and due to its common upregulation in colorectal cancer tissues has been proposed as an early diagnostic marker, as validated in a recent meta-analysis study [129]. It acts as a potent downregulator of PTEN, which normally inhibits Akt phosphorylation. Thus, miR-21 overexpression stimulates cell proliferation, as inferred by studies in hepatocellular carcinoma [130], bladder cancer [131], and lung adenocarcinoma, where it also mediates chemoresistance to 5-fluorouracil [132]. In addition, SW480 CRC cell line-secreted exosomal miR-21-5p induces angiogenesis and vascular permeability by KRIT1 suppression and β-catenin activation in recipient endothelial cells [133], while M2 macrophage-derived exosomes containing miR-21-5p enhance CRC cell migration and invasion in the SW48 and SW480 cell lines and in vivo [134]. Interestingly, however, it has been reported that miR-21-5p can induce pyroptosis in HCT116 and HT29 cell lines—a form of programmed cell death associated with inflammation—by downregulating TGFBI, a prominent EMT marker [110].

Another key factor to take into account is the potential feedback between adiponectin signaling and miRNA expression. Adiponectin and its receptors, which play a critical role in metabolic and inflammatory regulation, could themselves influence the biogenesis of miRNAs. Adiponectin has been shown to inhibit LPS-induced pyroptosis in nucleus pulposus cells through the upregulation of miR-135a-5p [135], while in esophageal adenocarcinoma it exhibits the opposite effect—induction of pyroptosis via upregulation of miR-378a-3p [136]. Adiponectin also upregulates miR-133a in a rat model of cardiac hypertrophy [137] and in a mouse model of acute aortic dissection, where it also inhibits pyroptosis [138]. These data suggest a bidirectional regulatory axis where miRNAs not only control adiponectin pathways but also respond to dysregulation in adiponectin levels characteristic for obesity.

One of the most intriguing findings of our study was that miRNAs regulating APPL1, a protein mediating adiponectin’s effects on Akt, and p38 MAPK signaling pathways, are consistently dysregulated in CRC. These pathways are crucial for metabolic homeostasis and inflammation, suggesting that miRNAs disrupting APPL1 expression interfere with downstream adiponectin signaling. Moreover, given the established interaction between adiponectin signaling and AMPK activation, the role of p38 MAPK in this context further strengthens the link between impaired adiponectin signaling and CRC progression. As p38 activation is thought to be AMPK-dependent, the disruption of these pathways by miRNAs could contribute to a cancer-promoting environment by weakening metabolic and growth control mechanisms in epithelial cells.

A notable limitation of our current in silico study is the lack of differentiation between the established CRC subtypes due to the absence of distinction between them when performing meta-profiling of large numbers of complex datasets. Moreover, the same analysis could be performed using a more lenient cut-off and an expanded subset of adiponectin activation cluster genes, which would extend the list of miRNAs hits. Given the preliminary nature of the current analyses, these considerations should be address in future work, especially with the exciting upcoming updates of the utilized databases.

## 4. Materials and Methods

The present study combines bioinformatics analysis of several publicly available miRNA databases and a literature review of dysregulated miRNAs in CRC patients’ tissues and CRC cell lines reported over the past twenty years.

### 4.1. Data Mining and Presentation

To assess the interplay between miRNAs, adiponectin signaling, and CRC oncogenesis, we adopted a cross-database mining approach, resulting in the identification of several specific miRNAs as candidates for future experimental validation. All recorded instances of individual miRNAs dysregulated in CRC vs. normal tissue transcriptomics datasets were extracted from the miRCancer database (hosted by the East Carolina University, Greenville, North Carolina, United States) [139], then validated and selected through the meta-profiling feature of the dbDEMC 3.0 database (hosted by the Fudan University, Yangpu, Shanghai, China) [140], based on a vote-counting strategy (VC) score, accounting for the average miRNA fold-change, number of studies reporting differential expression, and total amount of samples used across multiple datasets [141]. No additional FDR analysis or data corrections were performed, as only statistically significant entries with FDR < 0.05 have been deposited on the dbDEMC 3.0 database [140]. For the miRNAs of interest, gene ontologies were constructed for all the genes targeted by them, as inferred by miRTarBase (hosted by the Chinese University of Hong Kong, Hong Kong, China) [142], in order to discern which pathways are enhanced by the downregulation of miRNAs targeting the adiponectin axis, and which are silenced through the upregulation of miRNAs. The ontology networks were constructed with the CytoScape software (created at the Institute of Systems Biology, Seattle, Washington, DC, USA) version 3.10.3 [143], using the ClueGO application [144] and the WikiPathways [145] (hosted by the Maastricht University, Maastricht, The Netherlands) database under very stringent criteria for assignment of genes to pathways—*p* values cutoff set to 0.001, clustering cutoff set to at least 5 genes participating in a pathway, false-discovery rate cutoff set to 0.25 with a connectivity (kappa) score set to above 0.4. Additionally, we used MiRNET’s (hosted by McGill University, Montreal, QC, Canada) [146] miRNA query feature to pinpoint and visualize which of the genes added to the ontology are affected by more than one miRNA, i.e., would be most impacted by the miRNA dysregulation. The degree cutoff was set at 2.0, so that each resulting gene is linked to at least three miRNAs.

### 4.2. Data Source and Literature Search

PubMed, Web of Science, and Google Scholar were searched between May and December 2024 for English full-text articles (2004–2024) on the correlation between dysregulated miRNAs and CRC in patients and cell lines. Keywords included “colorectal cancer,” “obesity,” “adiponectin signaling,” and “miRNAs,” focusing on experimentally validated miRNA-mediated mechanisms in CRC and their link to adiponectin signaling. The overall study design is illustrated in Supplementary Figure S1.

## 5. Conclusions

In summary, bioinformatics methods identified seven miRNAs (hsa-miR-215-5p, hsa-miR-340-5p, hsa-miR-181a-5p, hsa-miR-150-5p, hsa-miR-96-5p, hsa-miR-19a-3p, and hsa-miR-21-5p) that affect upstream adiponectin signaling and are systematically altered in CRC. They were shown to target specific biological processes and pathways involved in tumor suppression or progression, as revealed by GO enrichment analysis, providing insight into their contributions to malignancy. Presently, neither of the selected miRNAs have been reported to impact adiponectin signaling in the context of CRC. The described bioinformatics workflow constitutes an exciting tool allowing for the identification of promising miRNAs from large datasets and can be readily adapted to various queries. Nevertheless, in vitro studies are imperative to assess the proposed wide applicability of such in silico analysis. Quantitative experiments with the miRNA “hits” presented herein are currently being performed to elucidate their exact roles in the intricate network of CRC pathogenesis.

## Figures and Tables

**Table 1:** List for the final selection of miRNAs based on our analysis workflow, their target genes from the adiponectin pathway, and the miRNAs’ dysregulation profile.

miRNA	Target Genes from Pathway	Profile	VC Score
hsa-miR-215-5p	APPL1	downregulated	−5
hsa-miR-340-5p	APPL1/AKT1	downregulated	−4
hsa-miR-181a-5p	CDH13	downregulated	−4
hsa-miR-150-5p	PRKAB1/ADIPOR2	downregulated	−8
hsa-miR-96-5p	APPL1/IRS1/AKT1	upregulated	13
hsa-miR-19a-3p	PPARA/PRKAA1/AKT1/ADIPOR2	upregulated	9
hsa-miR-21-5p	PPARA/PRKAB2/APPL1	upregulated	16

APPL1, Adaptor Protein, Phosphotyrosine Interacting with PH Domain and Leucine Zipper 1; AKT1, AKT Serine/Threonine Kinase 1; CDH13, T-Cadherin; PRKAB1, Protein Kinase AMP-Activated Non-Catalytic Subunit Beta 1; ADIPOR2, Adiponectin Receptor 2; IRS1, Insulin Receptor Substrate 1; PPARA, Peroxisome Proliferator-Activated Receptor Alpha; PRKAA1, Protein Kinase AMP-Activated Catalytic Subunit Alpha 1; PRKAB2, Protein Kinase AMP-Activated Non-Catalytic Subunit Beta 2.

## Data Availability

No new data were created or analyzed in this study. Data sharing is not applicable to this article.

## Supplementary Materials

The following supporting information can be downloaded at: https://www.mdpi.com/article/10.3390/ijms26157196/s1.
