# Supplementary material for: Dysregulated miRNAs Targeting Adiponectin Signaling in Colorectal Cancer
Source: Int J Mol Sci. 2025 Jul 25;26(15):7196. doi: 10.3390/ijms26157196 (PMC12346623; doi:10.3390/ijms26157196)
Supplement: Supplementary file 1 [file ijms-26-07196-s001.zip › Supplemenary Tables S1,S2,S3,S4.pdf]

Supplementary Table S1. miRNAs with oncogenic potential, expressed in CRC patients' tissues.

miRNA	Expression pattern in CRC	Functional role in CRC	Ref.
miR-21	significantly upregulated	Promotes proliferation, migration, and invasion of CRC cells; High expression is significantly correlated with lymph node and distant metastases	[1] [2] [3]
miR-31	significantly upregulated	Promotes proliferation, migration, and invasion of CRC cells	[1] [4]
miR-32	significantly upregulated	Stimulates CRC lymphatic invasion, metastasis, and correlates with patients' poor survival.	[5]
miR-1290	significantly upregulated	Promotes tumor progression	[6] [7]
miR-888	significantly upregulated	Promotes cell proliferation, invasion and metastasis; overexpression results in decreased overall survival	[8]
miR-155-5p	upregulated	Promotes cell proliferation, invasion and metastasis in colorectal carcinoma	[9]
miR-325-3p	significantly upregulated	Promotes epithelial-to-mesenchymal transition; enhances migration and invasion of CRC cells	[10]
miR-27a	significantly upregulated	Promotes proliferation, migration, and invasion of colorectal cancer by targeting FAM172A	[11]
miR-20a	upregulated	High expression is significantly correlated with lymph node and distant metastases	[12]
miR-106a	significantly upregulated	Promotes cell proliferation and migration	[13]
miR-942	significantly upregulated	Promotes the Wnt - signaling activity	[14]
miR-135a	significantly upregulated	Promotes the growth and invasion of CRC cells through targeting MTSS1.	[15]
miR-135b	significantly upregulated	Downregulates MTSS1 expression and contributes to CRC cell invasion; promotes lymph nodes and distant metastasis.	[16]
miR-182	significantly upregulated	Contributes to cell proliferation, invasion and tumor growth in colorectal cancer by targeting DAB2IP	[17]
miR-103a-3p	significantly upregulated	Promotes the proliferation and metastasis of colon cancer cells	[18] [19]
miR-18a-5p	significantly upregulated	Promotes the proliferation and metastasis of colon cancer cells	[19]

miR-127-3p	significantly upregulated	Promotes the proliferation and metastasis of colon cancer cells	[19]
miR-17	significantly upregulated	Promotes cell proliferation, tumor growth and cell cycle progression by targeting the RND3 tumor suppressor gene	[20] [21] [22]
miR-17-5p	significantly upregulated	Promotes the proliferation and metastasis of colon cancer cells	[19]
miR-19a	upregulated	Mediates the TNF-α induced epithelial-to-mesenchymal transition in CRC; Overexpression is associated with lymph metastasis	[23]
miR-19a-3p	significantly upregulated	Promotes the proliferation and metastasis of colon cancer cells	[3]
miR-425-5p	significantly upregulated	Promotes the proliferation and metastasis of colon cancer cells	[3]
miR-200c-3p	significantly upregulated	Enhances cell proliferation, invasion and tumor growth; contributes to poor overall survival	[24]
miR-592	significantly upregulated	Promotes cell proliferation, migration and invasion in colorectal cancer by directly targeting SPARC	[25]
miR-96	significantly upregulated	Promotes growth, proliferation, and survival of cancer cells	[26]
miR-181a	upregulated	Promotes tumor growth and liver metastasis in colorectal cancer by targeting the tumor suppressor WIF-1 (Wnt inhibitory factor)	[27]
miR-26a	upregulated	Enhances colorectal cancer cell growth by targeting RREB1 deacetylation to activate AKT-mediated glycolysis	[28]

CRC, Colorectal Cancer; FAM172A, Family with Sequence Similarity 172 Member A; WIF-1, Wnt Inhibitory Factor 1; RREB1, Ras Responsive Element Binding Protein 1; AKT, Protein Kinase B; EMT, Epithelial-to-Mesenchymal Transition; SPARC, Secreted Protein Acidic and Rich in Cysteine; MTSS1, Metastasis Suppressor 1; TGFBR2, Transforming Growth Factor Beta Receptor 2

Supplementary Table S2. miRNAs tumor-suppressive role, expressed in CRC patient’s tissues.

miRNA	Expression pattern in CRC	Functional role in CRC	Ref.
Let-7	downregulated	Directly downregulates some oncoproteins such as MYC, HMGA2, and IGF1 - known as critical regulators for the growth of CRC.	[29]
miR-202-5p	significantly downregulated	Inhibits CRC cell growth, migration, invasion and colony formation, and promotes cell apoptosis by targeting 3′-UTR of SMARCC1	[30]
miR-143-3p	significantly downregulated	Inhibits invasion and migration of CRC cells by downregulating ITGA6/ASAP3 expression	[31]
miR-34a	significantly downregulated	Suppresses cell proliferation by modulating the E2F signaling pathway	[32]
miR-22	significantly downregulated	Suppresses tumor invasion and metastasis in CRC;<br>inhibits autophagy in CRC;<br>Functions as a switch by targeting BTG1 from autophagy to apoptosis.<br><br>Suppresses tumor invasion and metastasis in CRC by targeting NLRP3	[33]<br><br>[34]<br><br>[35]
miR-145	significantly downregulated in metastatic tumors	Suppresses oncogene c-Myc;<br><br>Suppresses colon cancer invasion and metastasis by targeting LASP1	[36]<br><br>[37]
miR-141-3p	significantly downregulated	Inhibits cell proliferation, migration and invasion by targeting TRAF5	[38]
miR-141-3p	significantly downregulated	Inhibits CRC by regulating Bcl2	[39]
miR-1-3p	downregulated	Suppresses CRC proliferation and metastasis by inhibiting YWHAZ-Mediated Epithelial–Mesenchymal Transition	[40]
miR-215-5p	significantly downregulated	Reduces proliferation, formation of new colonies, and migration and increases apoptosis.	[41]
miR-150-5p	downregulated	Suppresses tumor progression by targeting VEGFA	[42]
miR-340-5p	significantly downregulated	Inhibitory effect on proliferation, migration and invasion of CRC	[43]

miR-325	significantly downregulated	Sensitizes CRC cells to oxaliplatin-induced cytotoxicity	[44]
miR-181a	downregulated	Inhibits cancer cell migration and angiogenesis by downregulating matrix metalloproteinase-14 expression.	[45]

MYC, Myelocytomatosis Oncogene; HMGA2, High Mobility Group AT-Hook 2; IGF1, Insulin-Like Growth Factor 1; SMARCC1, SWI/SNF-Related, Matrix-Associated, Actin-Dependent Regulator of Chromatin Subfamily C Member 1; ITGA6, Integrin Subunit Alpha 6; ASAP3, ArfGAP with SH3 Domain, Ankyrin Repeat, and PH Domain 3; E2F, E2F Transcription Factor; BTG1, B-Cell Translocation Gene 1; NLRP3:, NOD-, LRR-, and Pyrin Domain-Containing Protein 3; LASP1, LIM And SH3 Protein 1; TRAF5, TNF Receptor-Associated Factor 5; YWHAZ: Tyrosine 3-Monooxygenase/Tryptophan 5-Monooxygenase Activation Protein Zeta; VEGFA, Vascular Endothelial Growth Factor A; ANXA3, Annexin A3

Supplementary Table S3. miRNAs with oncogenic potential in human CRC-derived cell lines.

miRNA	Cell line	Role in CRC	Ref.
miR-1290	Caco2; DLD1;HT29; LoVo; SW480	Promotes tumor progression in CRC cell lines	[7] [6]
miR-888	SW620	Significantly promotes proliferation, migration and invasion of CRC cell lines by targeting the Smad4 signaling	[8]
hsa-miR-21	HT-29; SW-480; DLD-1	Stimulates proliferation, migration, and invasion of CRC cells; promotes tumor growth by inhibition of Sec23A expression in CRC cell lines	[46]
miR-32	LOVO, HT-29, HCT-116, SW480, SW620	Promotes CRC cells growth, migration, and invasion and reduces apoptosis in vitro; overexpression of miR-32 suppresses PTEN / anti-oncogene/ protein levels	[47]
miR-325-3p	HT-29	Stimulates migration and invasion of CRC cells;	[10]
miR-27a	LoVo, HT-29, SW480, HCT116, DLD-1 and RKO	Promotes cell proliferation, migration and invasion in CRC by regulation of FAM172A .	[11]
miR-20a	SW480	Enhances cell invasion and migration; high expression is correlated with lymph node metastases and distant metastases	[12]
miR-182	SW480, LoVo, HCT116, Caco-2 and SW620	Contributes to cell proliferation, invasion and tumor growth in CRC by targeting DAB2IP	[17]
miR-19	SW480 and SW620	Enhances invasion and metastasis by targeting transglutaminase-2	[48]

miR-19a-3p	HT29, SW480, SW620, and HCT116	Promotes cell proliferation by regulating the Forkhead box F2-mediated Wnt/β-catenin signaling pathway	[49]
miR-425-5p	SW480 and LoVo	Promotes tumor growth and metastasis by activating the CTNND1-mediated β-catenin pathway and EMT	[50]
miR-106a	SW620; HT29	Promotes cell proliferation and migration	[13]
miR-942	SW480	Promotes cell cycle progression	[14]
miR-592	HT29 and SW480	Enhances migratory and invasive abilities of CRC cells	[25]
miR– 155-5p	HT-29	Promotes proliferation, invasion and metastasis of CRC cells.	[9]
miR– 155	HCT116	Suppresses the mRNA and protein levels of PTPRJ (known for its antiproliferative role)	[51]
miR-135a	SW480 and SW620 CRC	Increases proliferation; promotes mobility and invasion of CRC cells through targeting MTSS1.	[15]
miR-135b	HT-29 and SW-480	Promotes the proliferation and inhibits the apoptosis of CRC cells by targeting TGFBR2	[16]

Caco2, DLD1, HT29, LoVo, SW480, SW620, Colorectal cancer cell lines; Smad4, Mothers Against Decapentaplegic Homolog 4; Sec23A, Sec23 Homolog A, COPII Coated Vesicle Component; PTEN, Phosphatase and Tensin Homolog; MTSS1, Metastasis Suppressor 1; TGFBR2, Transforming Growth Factor Beta Receptor 2; CTNND1, Catenin Delta 1

Supplementary Table S4. miRNAs with tumor-suppressive role in human CRC-derived cell lines.

miRNA	Cell line	Role in CRC	Ref.
miR-4443	HCT116; HT29	Reduces proliferation and invasion of CRC cells	[52]
miR-34a	HCT116; RKO	Suppresses cell proliferation by downregulation of the E2F signaling pathway and upregulation of the p53 signaling pathway.	[32]
	WT	Inhibits SIRT1 expression	[53]
	SW480		[54]

		Inhibits migration and invasion by regulating the SIRT1/p53 pathway	
miR-141-3p	SW480	miR-141–3p overexpression significantly attenuates proliferation, migration and invasion of CRC cells	[39]
	SW620, HCT116, SW480, Caco2 and HT29	Inhibits CRC cell proliferation, migration and invasion; miR-141–3p overexpression markedly suppresses proliferation of HCT116 and SW480 cells Inhibits CRC cell proliferation, migration and invasion; miR-141–3p overexpression markedly suppresses proliferation of HCT116 and SW480 cells	[38]
	HCT15, SW48	miR-141-3p inhibits growth of CRC cells and enhances cetuximab sensitivity	[55]
miR-1-3p	HT29, HCT116, SW480	Suppresses Colorectal Cancer Cell Proliferation and Metastasis by inhibiting YWHAZ-Mediated Epithelial–Mesenchymal Transition	[40]
miR-22	HCT116	Significantly decreases proliferation, migration, and invasion of HCT116 cells	[35]
miR-202-5p	DLD1, HCT116, LOVO, RKO, LS174T, HCT8, HR28348, HT29, SW620; SW480	Overexpression of miR-202-5p inhibits cell growth, cell migration and invasion, and induces cell apoptosis through downregulation of SMARCC1	[30]
miR-215-5p	HCT-116$^{+/+}$, DLD-1, HT-29, CaCo2 , HCT-116$^{-/-}$	Higher levels of miR-215-5p significantly reduce metabolic activity and proliferation of CRC cell lines.	[41]
miR-145	HCT116, HT29, LOVO, SW480; SW620	Inhibits the invasion and metastasis of CRC cells	[37]
miR-340-5p	SW480, HCT116, LOVO; HT-29	Exerts inhibitory effect on migration and invasion of CRC through targeting ANXA3	[43]
hsa-miR-21-5p	HCT-116; HT-29	Induces pyroptosis in CRC via TGFBI	[56]

E2F, E2F Transcription Factor; p53, Tumor Protein 53; SIRT1, Sirtuin 1; ANXA3, Annexin A3; TGFBI, Transforming Growth Factor Beta Induced

REFERENCES:

1. Slaby, O.; Svoboda, M.; Fabian, P.; Smerdova, T.; Knoflickova, D.; Bednarikova, M.; Nenutil, R.; Vyzula, R. Altered expression of miR-21, miR-31, miR-143 and miR-145 is related to clinicopathologic features of colorectal cancer. *Oncology* **2007**, *72* (5-6), 397-402. https://doi.org/10.1159/000113489.
2. Toiyama, Y.; Takahashi, M.; Hur, K.; Nagasaka, T.; Tanaka, K.; Inoue, Y.; Kusunoki, M.; Boland, C. R.; Goel, A. Serum miR-21 as a diagnostic and prognostic biomarker in colorectal cancer. *J. Natl. CancerInst.* **2013**, *105* (12), 849-859. https://doi.org/10.1093/jnci/djt101.
3. Zhu, M.; Huang, Z.; Zhu, D.; Zhou, X.; Shan, X.; Qi, L. W.; Wu, L.; Cheng, W.; Zhu, J.; Zhang, L.; Zhang, H.; Chen, Y.; Zhu, W.; Wang, T.; Liu, P. A panel of microRNA signature in serum for colorectal cancer diagnosis. *Oncotarget* **2017**, *8* (10), 17081-17091. https://doi.org/10.18632/oncotarget.15059.
4. Sarver, A. L.; French, A. J.; Borralho, P. M.; et al. Human colon cancer profiles show differential microRNA expression depending on mismatch repair status and are characteristic of undifferentiated proliferative states. *BMC Cancer* **2009**, *9*, 401. https://doi.org/10.1186/1471-2407-9-401.
5. Chen, E.; Li, Q.; Wang, H.; Zhang, P.; Zhao, X.; Yang, F.; Yang, J. MiR-32 promotes tumorigenesis of colorectal cancer by targeting BMP5. *Biomed. Pharmacother.* **2018**, *106*, 1046-1051. https://doi.org/10.1016/j.biopha.2018.07.050.
6. Wu, J.; Ji, X.; Zhu, L.; Jiang, Q.; Wen, Z.; Xu, S.; Shao, W.; Cai, J.; Du, Q.; Zhu, Y.; Mao, J. Up-regulation of microRNA-1290 impairs cytokinesis and affects the reprogramming of colon cancer cells. *Cancer Lett.* **2013**, *329* (2), 155-163. https://doi.org/10.1016/j.canlet.2012.10.038.
7. Imaoka, H.; Toiyama, Y.; Fujikawa, H.; Hiro, J.; Saigusa, S.; Tanaka, K.; Inoue, Y.; Mohri, Y.; Mori, T.; Kato, T.; Toden, S.; Goel, A.; Kusunoki, M. Circulating microRNA-1290 as a novel diagnostic and prognostic biomarker in human colorectal cancer. *Ann. Oncol.* **2016**, *27* (10), 1879-1886. https://doi.org/10.1093/annonc/mdw279.
8. Gao, S.; Chen, L.; Lu, W.; Zhang, L.; Wang, L.; Zhu, H. miR-888 functions as an oncogene and predicts poor prognosis in colorectal cancer. *Oncol. Lett.* **2018**, *15*, 9101-9109. https://doi.org/10.3892/ol.2018.8461.
9. Qu, Y. L.; Wang, H. F.; Sun, Z. Q.; Tang, Y.; Han, X. N.; Yu, X. B.; Liu, K. Up-regulated miR-155-5p promotes cell proliferation, invasion and metastasis in colorectal carcinoma. *Int. J. Clin. Exp. Pathol.* **2015**, *8* (6), 6988-6994. PMID: 26261588; PMCID: PMC4525922.
10. Song, C.; Wang, X.; Zhao, X.; Ai, J.; Qi, Y.; Chen, A. MicroRNA 325-3p contributes to colorectal carcinoma by targeting cytokeratin 18. *Oncol. Lett.* **2021**, *21*, 248. https://doi.org/10.3892/ol.2021.12509.
11. Liu, W.; Qian, K.; Wei, X.; Deng, H.; Zhao, B.; Chen, Q.; Liu, H. miR-27a promotes proliferation, migration, and invasion of colorectal cancer by targeting FAM172A and acts as a diagnostic and prognostic biomarker. *Oncol. Rep.* **2017**, *37*, 3554-3564. https://doi.org/10.3892/or.2017.5592.
12. Zhang, G.; Li, Y.; Zhou, H.; Xiao, H.; Zhou, T. miR-20a is an independent prognostic factor in colorectal cancer and is involved in cell metastasis. *Mol. Med. Rep.* **2014**, *10*, 283-291. https://doi.org/10.3892/mmr.2014.2144.
13. Qin, Y.; Chen, X.; Liu, Z.; Tian, X.; Huo, Z. miR-106a reduces 5-fluorouracil (5-FU) sensitivity of colorectal cancer by targeting dual-specificity phosphatases 2 (DUSP2). *Med.*

Sci. Monit. **2018**, *24*, 4944-4951. https://doi.org/10.12659/MSM.910016. PMID: 30011263; PMCID: PMC6067019.
14. Fasihi, A.; Soltani, B. M.; Ranjbaran, Z. S.; Bahonar, S.; Norouzi, R.; Nasiri, S. Hsa-miR-942 fingerprint in colorectal cancer through Wnt signaling pathway. *Gene* **2019**, *712*, 143958. https://doi.org/10.1016/j.gene.2019.143958.
15. Zhou, W.; Li, X.; Liu, F.; Xiao, Z.; He, M.; Shen, S.; Liu, S. MiR-135a promotes growth and invasion of colorectal cancer via metastasis suppressor 1 in vitro. *Acta Biochim. Biophys. Sin.* **2012**, *44* (10), 838-846. https://doi.org/10.1093/abbs/gms071.
16. Li, J.; Liang, H.; Bai, M.; Ning, T.; Wang, C.; Fan, Q.; et al. Correction: miR-135b promotes cancer progression by targeting transforming growth factor beta receptor II (TGFBR2) in colorectal cancer. *PLoS ONE* **2015**, *10* (12), e0145589. https://doi.org/10.1371/journal.pone.0145589.
17. Li, X.; Zhang, X.; Zhang, Q.; Lin, R. MiR-182 contributes to cell proliferation, invasion, and tumor growth in colorectal cancer by targeting DAB2IP. *Int. J. Biochem. CellBiol.* **2019**, *111*, 27-36. https://doi.org/10.1016/j.biocel.2019.04.002.
18. Chen, H.-Y.; Lin, Y.-M.; Chung, H.-C.; Lang, Y.-D.; Lin, C.-J.; Huang, H.-D.; et al. miR-103/107 promote metastasis of colorectal cancer by targeting the metastasis suppressors DAPK and KLF4. *Cancer Res.* **2012**, *72* (14), 3631-3641. https://doi.org/10.1158/0008-5472.CAN-12-0667.
19. Zhang, H.; Zhu, M.; Shan, X.; Zhou, X.; Wang, T.; Zhang, J.; et al. A panel of seven-miRNA signature in plasma as potential biomarker for colorectal cancer diagnosis. *Gene* **2019**, *687*, 246-254. https://doi.org/10.1016/j.gene.2018.11.055.
20. Luo, H.; Zou, J.; Dong, Z.; Zeng, Q.; Wu, D.; Liu, L. Up-regulated miR-17 promotes cell proliferation, tumour growth and cell cycle progression by targeting the RND3 tumour suppressor gene in colorectal carcinoma. *Biochem. J.* **2012**, *442* (2), 311-321. https://doi.org/10.1042/BJ20111517.
21. Chen, X.; Shi, K.; Wang, Y.; Song, M.; Zhou, W.; Tu, H.; Lin, Z. Clinical value of integrated-signature miRNAs in colorectal cancer: miRNA expression profiling analysis and experimental validation. *Oncotarget* **2015**, *6* (35), 37544-37556. https://doi.org/10.18632/oncotarget.6065.
22. Pellatt, D. F.; Stevens, J. R.; Wolff, R. K.; Mullany, L. E.; Herrick, J. S.; Samowitz, W.; Slattery, M. L. Expression profiles of miRNA subsets distinguish human colorectal carcinoma and normal colonic mucosa. *Clin. Transl. Gastroenterol.* **2016**, *7* (3), e152. https://doi.org/10.1038/ctg.2016.11.
23. Huang, L.; Wang, X.; Wen, C.; Yang, X.; Song, M.; Chen, J.; Wang, C.; Zhang, B.; Wang, L.; Iwamoto, A.; et al. Hsa-miR-19a is associated with lymph metastasis and mediates the TNF-α induced epithelial-to-mesenchymal transition in colorectal cancer. *Sci. Rep.* **2015**, *5*, 13350. https://doi.org/10.1038/srep13350.
24. Kang, E.; Jung, S. C.; Nam, S. K.; Park, Y.; Seo, S. H.; Park, K. U.; Oh, H. K.; Kim, D. W.; Kang, S. B.; Lee, H. S. Tissue miR-200c-3p and circulating miR-1290 as potential prognostic biomarkers for colorectal cancer. *Sci. Rep.* **2022**, *12* (1), 2295. https://doi.org/10.1038/s41598-022-06192-w.
25. Pan, Z.; Xie, R.; Song, W.; Gao, C. MicroRNA 592 promotes cell proliferation, migration and invasion in colorectal cancer by directly targeting SPARC. *Mol. Med. Rep.* **2021**, *23*, 261. https://doi.org/10.3892/mmr.2021.11900.

26. Rapti, S. M.; Kontos, C. K.; Papadopoulos, I. N.; Scorilas, A. High miR-96 Levels in Colorectal Adenocarcinoma Predict Poor Prognosis, Particularly in Patients without Distant Metastasis at the Time of Initial Diagnosis. *Tumor Biol.* **2016**, *37*, 11815–11824.
27. Ji, D.; Chen, Z.; Li, M.; Zhan, T.; Yao, Y.; Zhang, Z.; Xi, J.; Yan, L.; Gu, J. MicroRNA-181a Promotes Tumor Growth and Liver Metastasis in Colorectal Cancer by Targeting the Tumor Suppressor WIF-1. *Mol. Cancer* **2014**, *13*, 86. https://doi.org/10.1186/1476-4598-13-86. PMID: 24755295; PMCID: PMC4021214.
28. Chen, B.; Deng, Y. N.; Wang, X.; Xia, Z.; He, Y.; Zhang, P.; Syed, S. E.; Li, Q.; Liang, S. miR-26a Enhances Colorectal Cancer Cell Growth by Targeting RREB1 Deacetylation to Activate AKT-Mediated Glycolysis. *Cancer Lett.* **2021**, *521*, 1–13. https://doi.org/10.1016/j.canlet.2021.08.017. Epub ahead of print. PMID: 34419497.
29. Mizuno, R.; Kawada, K.; Sakai, Y. The molecular basis and therapeutic potential of Let-7 microRNAs against colorectal cancer. *Can. J. Gastroenterol. Hepatol.* **2018**, Article ID 5769591, 7 pages. https://doi.org/10.1155/2018/5769591.
30. Ke, S.-B.; Qiu, H.; Chen, J.-M.; Shi, W.; Chen, Y.-S. MicroRNA-202-5p functions as a tumor suppressor in colorectal carcinoma by directly targeting SMARCC1. *Gene* **2018**, *676*, 329-335. https://doi.org/10.1016/j.gene.2018.08.064.
31. Guo, L.; Fu, J.; Sun, S.; et al. MicroRNA-143-3p inhibits colorectal cancer metastases by targeting ITGA6 and ASAP3. *CancerSci.* **2019**, *110*, 805-816. https://doi.org/10.1111/cas.13910.
32. Tazawa, H.; Tsuchiya, N.; Izumiya, M.; Nakagama, H. Tumor-suppressive miR-34a induces senescence-like growth arrest through modulation of the E2F pathway in human colon cancer cells. *Proc. Natl. Acad. Sci. U.S.A.* **2007**, *104* (39), 15472-15477.
33. Zhang, G.; Xia, S.; Tian, H.; et al. Clinical Significance of miR-22 Expression in Patients with Colorectal Cancer. *Med. Oncol.* **2012**, *29*, 3108–3112. https://doi.org/10.1007/s12032-012-0233-9.
34. Zhang, H.; Tang, J.; Li, C.; Kong, J.; Wang, J.; Wu, Y.; Xu, E.; Lai, M. MiR-22 regulates 5-FU sensitivity by inhibiting autophagy and promoting apoptosis in colorectal cancer cells. *CancerLett.* **2015**, *356* (2), 781-790. https://doi.org/10.1016/j.canlet.2014.10.029.
35. Cong, J.; Gong, J.; Yang, C.; Xia, Z.; Zhang, H. MiR-22 suppresses tumor invasion and metastasis in colorectal cancer by targeting NLRP3. *Cancer Manag. Res.* **2020**, *12*, 5419-5429. https://doi.org/10.2147/CMAR.S255125.
36. Sachdeva, M.; Mo, Y.-Y. MiR-145-mediated suppression of cell growth, invasion and metastasis. *Am. J. Transl. Res.* **2010**, *2* (2), 170-180. PMID: 20407606; PMCID: PMC2855636.
37. Wang, W.; Ji, G.; Xiao, X.; Chen, X.; Qin, W. W.; Yang, F.; Li, Y. F.; Fan, L. N.; Xi, W. J.; Huo, Y.; et al. Epigenetically regulated miR-145 suppresses colon cancer invasion and metastasis by targeting LASP1. *Oncotarget* **2016**, *7* (42), 68674-68687. https://doi.org/10.18632/oncotarget.11919.
38. Liang, Z.; Li, X.; Liu, S.; Li, C.; Wang, X.; Xing, J. MiR-141-3p inhibits cell proliferation, migration and invasion by targeting TRAF5 in colorectal cancer. *Biochem. Biophys. Res. Commun.* **2019**, *514* (3), 699-705. https://doi.org/10.1016/j.bbrc.2019.05.002.
39. Tong, J.; Zhang, X. Y.; Guo, H. F.; et al. Study on effects of miR-141-3p in proliferation, migration, invasion and apoptosis of colon cancer cells by inhibiting Bcl2. *Clin. Transl. Oncol.* **2021**, *23*, 2526-2535. https://doi.org/10.1007/s12094-021-02653-2.

40. Du, G.; Yu, X.; Chen, Y.; Cai, W. MiR-1-3p suppresses colorectal cancer cell proliferation and metastasis by inhibiting YWHAZ-mediated epithelial–mesenchymal transition. *Front. Oncol.* **2021**, *11*, 634596.
41. Vychytilova-Faltejskova, P.; Merhautova, J.; Machackova, T.; Gutierrez-Garcia, I.; Garcia-Solano, J.; Radova, L.; Slaby, O. MiR-215-5p is a Tumor Suppressor in Colorectal Cancer Targeting EGFR Ligand Epiregulin and Its Transcriptional Inducer HOXB9. *Oncogenesis* **2017**, *6* (11), 399.
42. Chen, X.; Xu, X.; Pan, B.; Zeng, K.; Xu, M.; Liu, X.; ... Wang, S. MiR-150-5p suppresses tumor progression by targeting VEGFA in colorectal cancer. *Aging (Albany NY)* **2018**, *10* (11), 3421. https://doi.org/10.18632/aging.101656.
43. Yang, L.; Men, W. L.; Yan, K. M.; Tie, J.; Nie, Y. Z.; Xiao, H. J. MiR-340-5p is a Potential Prognostic Indicator of Colorectal Cancer and Modulates ANXA3. *Eur. Rev. Med. Pharmacol. Sci.* **2018**, *22* (15).
44. Zhang, L.; Chen, H.; Song, Y.; et al. MiR-325 Promotes Oxaliplatin-Induced Cytotoxicity Against Colorectal Cancer Through the HSPA12B/PI3K/AKT/Bcl-2 Pathway. *Dig. Dis. Sci.* **2021**, *66*, 2651–2660. https://doi.org/10.1007/s10620-020-06579-7.
45. Li, Y.; Kuscu, C.; Banach, A.; Zhang, Q.; Pulkoski-Gross, A.; Kim, D.; Liu, J.; Roth, E.; Li, E.; Shroyer, K. R.; Denoya, P. I.; Zhu, X.; Chen, L.; Cao, J. miR-181a-5p Inhibits Cancer Cell Migration and Angiogenesis via Downregulation of Matrix Metalloproteinase-14. *Cancer Res.* **2015**, *75*(13), 2674–2685. https://doi.org/10.1158/0008-5472.CAN-14-2875.
46. Li, C.; Zhao, L.; Chen, Y.; He, T.; Chen, X.; Mao, J.; ... Meng, Q. H. MicroRNA-21 Promotes Proliferation, Migration, and Invasion of Colorectal Cancer, and Tumor Growth Associated with Down-Regulation of Sec23a Expression. *BMC Cancer* **2016**, *16*, 1–11. https://doi.org/10.1186/s12885-016-2184-0.
47. Wu, W.; Yang, J.; Feng, X.; Wang, H.; Ye, S.; Yang, P.; Tan, W.; Wei, G.; Zhou, Y. MicroRNA-32 (miR-32) Regulates Phosphatase and Tensin Homologue (PTEN) Expression and Promotes Growth, Migration, and Invasion in Colorectal Carcinoma Cells. *Mol. Cancer* **2013**, *12* (1), 30. https://doi.org/10.1186/1476-4598-12-30.
48. Cellura, D.; Pickard, K.; Quaratino, S.; Parker, H.; Strefford, J. C.; Thomas, G. J.; ... Peake, N. J. MiR-19-mediated inhibition of transglutaminase-2 leads to enhanced invasion and metastasis in colorectal cancer. *Mol. CancerRes.* **2015**, *13* (7), 1095-1105. https://doi.org/10.1158/1541-7786.MCR-15-0097.
49. Yu, F. B.; Sheng, J.; Yu, J. M.; Liu, J. H.; Qin, X. X.; Mou, B. MiR-19a-3p regulates the Forkhead box F2-mediated Wnt/β-catenin signaling pathway and affects the biological functions of colorectal cancer cells. *World J. Gastroenterol.* **2020**, *26* (6), 627-644. https://doi.org/10.3748/wjg.v26.i6.627.
50. Liu, D.; Zhang, H.; Cui, M.; Chen, C.; Feng, Y. Hsa-miR-425-5p promotes tumor growth and metastasis by activating the CTNND1-mediated β-catenin pathway and EMT in colorectal cancer. *Cell Cycle* **2020**, *19* (15), 1917-1927. https://doi.org/10.1080/15384101.2020.1787307.
51. Zhang, X.-F.; Tu, R.; Li, K.; Ye, P.; Cui, X. Tumor suppressor PTPRJ is a target of miR-155 in colorectal cancer. *J. Cell. Biochem.* **2017**, *118* (10), 3391-3400. https://doi.org/10.1002/jcb.25995.
52. Meerson, A.; Yehuda, H. Leptin and Insulin Up-Regulate miR-4443 to Suppress NCOA1 and TRAF4 and Decrease the Invasiveness of Human Colon Cancer Cells. *BMC Cancer* **2016**, *16* (1), 882. https://doi.org/10.1186/s12885-016-2938-1.

53. Yamakuchi, M.; Ferlito, M.; Lowenstein, C. J. MiR-34a repression of SIRT1 regulates apoptosis. *Proc. Natl. Acad. Sci. U.S.A.* **2008**, *105* (36), 13421-13426. https://doi.org/10.1073/pnas.0801613105.
54. Lai, M.; Du, G.; Shi, R.; Yao, J.; Yang, G.; Wei, Y.; ... Wang, L. MiR-34a inhibits migration and invasion by regulating the SIRT1/p53 pathway in human SW480 cells. *Mol. Med. Rep.* **2015**, *11* (5), 3301-3307. https://doi.org/10.3892/mmr.2015.3146.
55. Xing, Y.; Jing, H.; Zhang, Y.; Suo, J.; Qian, M. MicroRNA-141-3p affected proliferation, chemosensitivity, migration and invasion of colorectal cancer cells by targeting EGFR. *Int. J. Biochem. Cell Biol.* **2020**, *118*, 105643. https://doi.org/10.1016/j.biocel.2019.105643.
56. Jiang, R.; Chen, X.; Ge, S.; Wang, Q.; Liu, Y.; Chen, H.; Xu, J.; Wu, J. MiR-21-5p induces pyroptosis in colorectal cancer via TGFBI. *Front. Oncol.* **2021**, *10*, 610545. https://doi.org/10.3389/fonc.2020.610545.
