# Supplementary material for: Dysregulated miRNAs Targeting Adiponectin Signaling in Colorectal Cancer
Source: Int J Mol Sci. 2025 Jul 25;26(15):7196. doi: 10.3390/ijms26157196 (PMC12346623; doi:10.3390/ijms26157196)
Supplement: Supplementary file 1 [file ijms-26-07196-s001.zip › Supplementary Figure S2.pptx]

## Slide 1
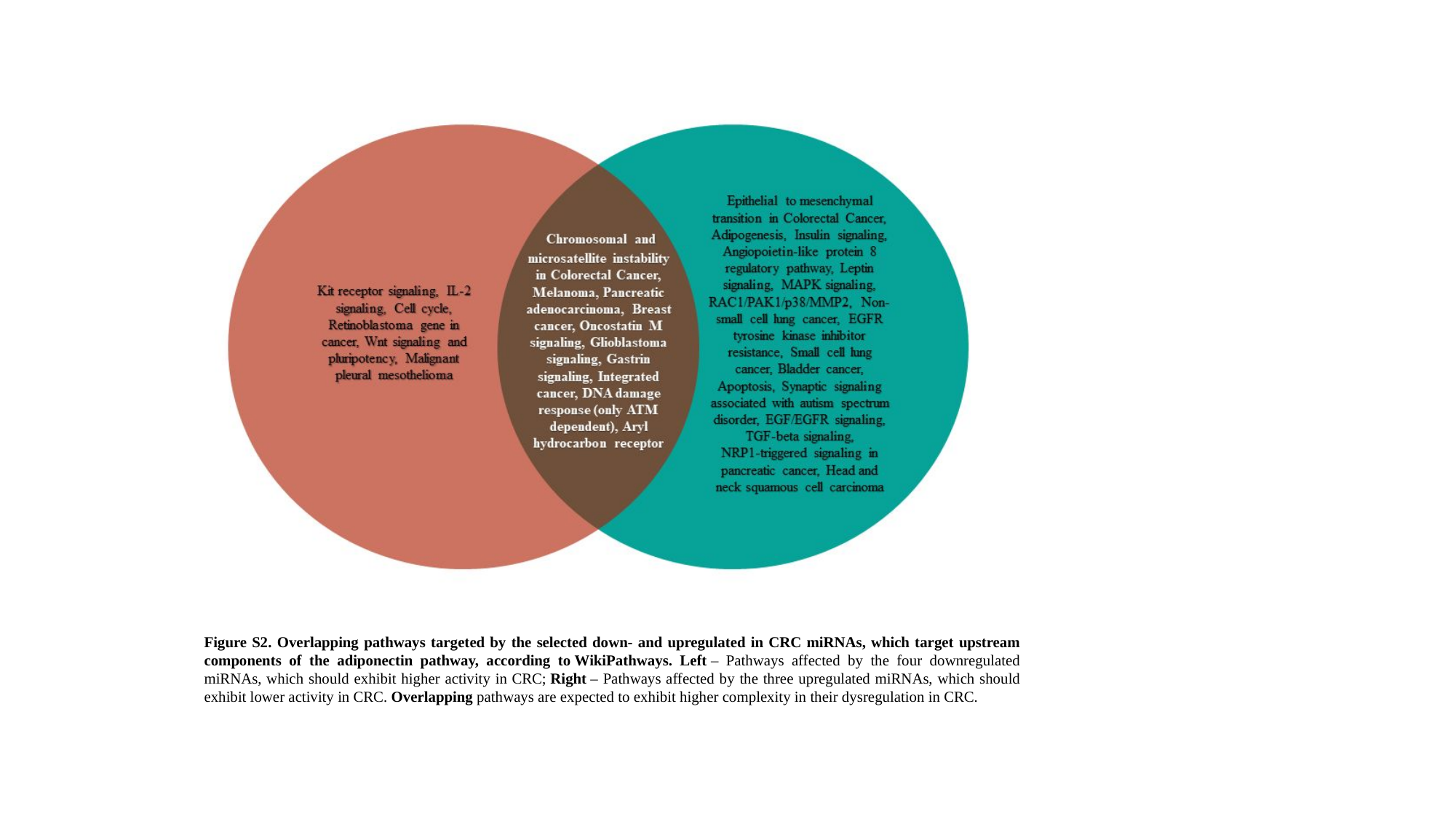

Figure S2. Overlapping pathways targeted by the selected down- and upregulated in CRC miRNAs, which target upstream components of the adiponectin pathway, according to WikiPathways. Left – Pathways affected by the four downregulated miRNAs, which should exhibit higher activity in CRC; Right – Pathways affected by the three upregulated miRNAs, which should exhibit lower activity in CRC. Overlapping pathways are expected to exhibit higher complexity in their dysregulation in CRC.
