# Supplementary material for: Dysregulated miRNAs Targeting Adiponectin Signaling in Colorectal Cancer
Source: Int J Mol Sci. 2025 Jul 25;26(15):7196. doi: 10.3390/ijms26157196 (PMC12346623; doi:10.3390/ijms26157196)
Supplement: Supplementary file 1 [file ijms-26-07196-s001.zip › SupplementaryFigure S1_07.05.25.pdf]

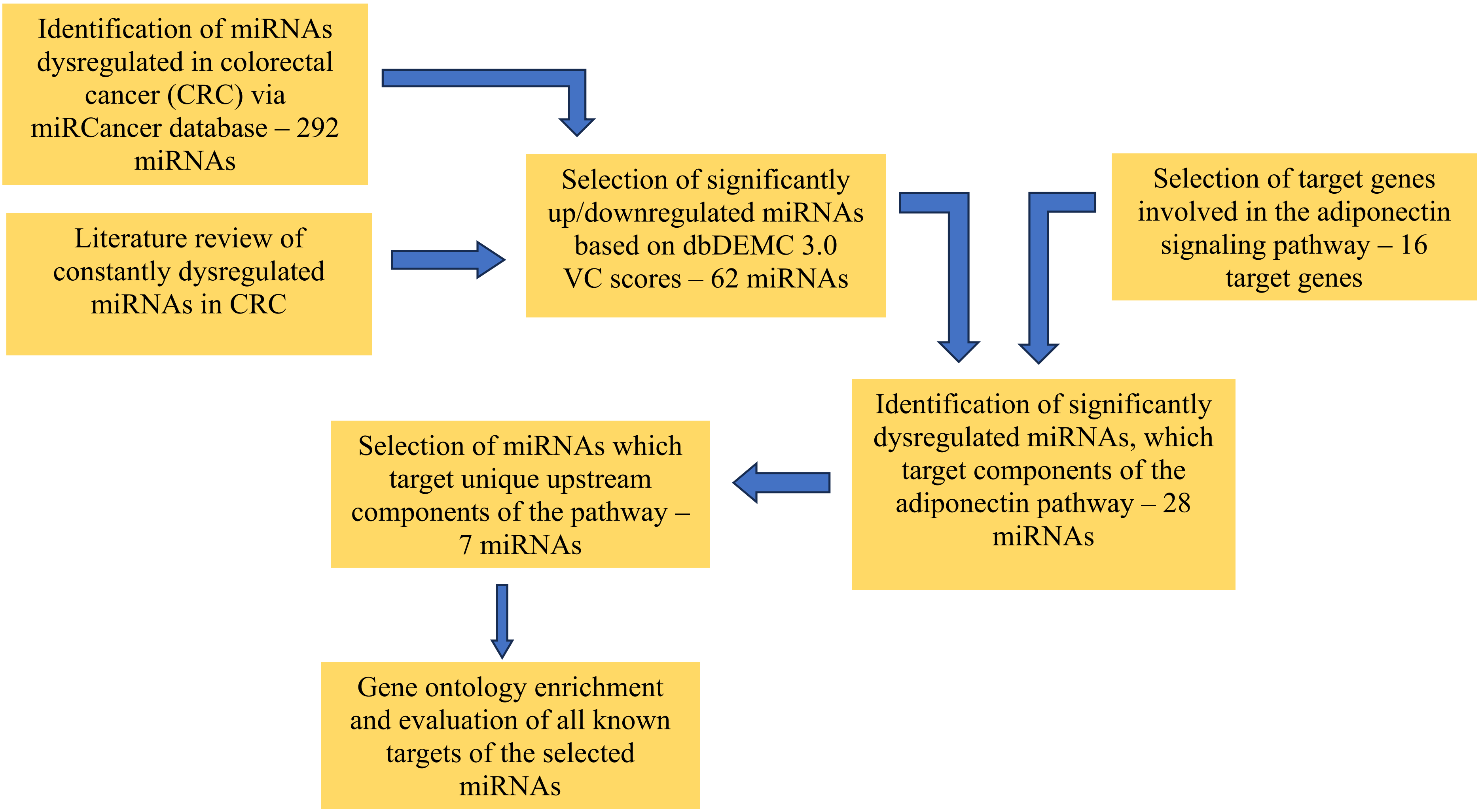

Figure S1. Study design scheme.

Workflow for bioinformatics analysis: Initially, 292 unique entries for dysregulated miRNAs in CRC were obtained and then cross-referenced with the dbDEMC 3.0. To reduce complexity and to obtain more concrete analysis results, only 62 miRNAs with VC scores of 4 and above (or -4 and below) were considered for the next steps of the workflow. Out of those, 28 were confirmed via miRTarBase to target selected mRNA transcripts for genes involved in the adiponectin pathway. From those 28, 7 were found to target upstream proteins - AdipoR1, AdipoR2, T-cadherin, and APPL1. Gene ontologies were then constructed for all genes targeted by the upregulated and downregulated selected miRNAs, respectively. ***Literature review:*** PubMed, Web of Science, and Google Scholar were searched between May and December 2024 for English full-text articles (2004–2024) on the correlation between dysregulated miRNAs and colorectal cancer (CRC) in patients and cell lines
