# Supplementary material for: Dysregulated miRNAs Targeting Adiponectin Signaling in Colorectal Cancer
Source: Int J Mol Sci. 2025 Jul 25;26(15):7196. doi: 10.3390/ijms26157196 (PMC12346623; doi:10.3390/ijms26157196)
Supplement: Supplementary file 1 [file ijms-26-07196-s001.zip › SupplementaryTables S5, S6.pdf]

Supplementary Table S5. The most-targeted genes by the four selected downregulated miRNAs (hsa-miR-215-5p; hsa-miR-340-5p; hsa-miR-181a-5p; hsa-miR-150-5p). These genes are expected to be significantly upregulated in CRC.

Gene	Characteristics of gene product	Reference
**SLC35G1**<br>Solute carrier family 35 member G1, also known as POST	Partner of STIM1. A transmembrane protein located on plasma and ER membranes involved in calcium homeostasis by interacting with STIM1 and modulating calcium pump ATPases. Shares structural features with some xenobiotic transporters, but no such function has been reported for it so far.	[1]
**DSN1**<br>Kinetochore-associated protein DSN1 homolog	Required for proper kinetochore assembly and chromosomal segregation during mitosis.<br>Its expression correlates with cancer progression and poor prognosis in colorectal cancer, hepatocellular carcinoma, and breast cancer.	[2]<br>[3]<br>[4]<br>[5]
**DEGS1**<br>Sphingosine Δ4 desaturase enzyme	Critical for neural function; impairment causes hypomyelinating leukodystrophy.<br>Associated with *de novo* ceramide synthesis pathway. Upregulation increases ceramide levels, linked to insulin resistance.	[6]<br>[7]
**GK5**<br>Glycerol kinase 5	An enzyme catalyzing the formation of glycerol-3-phosphate, involved in lipogenesis and skin cholesterol regulation. Its upregulation potentially leads to increased TAG synthesis due to higher concentration of the glycerol-3-phosphate precursor.<br>Its expression is elevated by cigarette smoke exposure, resulting in lipid droplet formation in pneumocytes.	[8]<br>[9]
**SLC7A11**<br>Solute carrier family 7 member A11, or xCT	A transmembrane antiporter exchanging extracellular cystine for intracellular glutamate, found as a heterodimer with SLC3A2, important for cellular glutathione homeostasis and redox balance.<br>Upregulated in many cancer types and contributes to tumor resistance to ferroptosis and chemotherapy, thus becoming a viable target for cancer treatment.	[10]<br>[11]<br>[12]
**XIAP**<br>X-linked inhibitor of apoptosis	An E3 ubiquitin ligase and caspase inhibitor. Functions include inhibition of cas-3, cas-7, and cas-9 and modulation of necroptosis, autophagy, inflammation, and copper homeostasis. Overexpression supports tumorigenesis and is highly expressed in various cancers.	[13]
**BCL2L11**<br>Bcl2-like protein 11 (BIM)	A pro-apoptotic protein inducing apoptosis in response to detachment from the extracellular matrix (anoikis). Functions as a tumor suppressor and is protective against oncogenesis when upregulated.	[14]
**ID4**<br>Inhibitor of DNA binding 4	Regulates gene expression by inhibiting bHLH transcription factors like c-Myc, HIF-1, and others.<br>Its role varies by cancer type: tumor-suppressive in colorectal and prostate cancer but oncogenic in breast cancer due to inhibition of BRCA1.	[15]<br>[16]<br>[17]<br>[18]

ER, Endoplasmic Reticulum; STIM1, Stromal Interaction Molecule 1; SCFAs, Short-Chain Fatty Acids; TAG, Triacylglycerol; xCT, Cystine/Glutamate Antiporter; cas-3, cas-7, cas-9, Caspases 3, 7, and 9; EGFR/MAPK, Epidermal Growth Factor Receptor/Mitogen-Activated Protein Kinase; bHLH, Basic Helix-Loop-Helix; HIF-1, Hypoxia-Inducible Factor 1; BRCA1, Breast Cancer Type 1 Susceptibility Protein.

Supplementary Table S6. The most-targeted genes by the three selected upregulated miRNAs (hsa-miR-96-5p; hsa-miR-19a-3p; hsa-miR-21-5p). These genes are expected to be significantly downregulated in CRC.

Gene	Characteristics of gene product	Reference
**MALT1**<br>Mucosa-associated lymphoid tissue lymphoma translocation protein 1	A paracaspase with oncogenic potential. Predominantly expressed in lymphocytes, forms a complex with BCL10, activating NF-κB-dependent survival pathways and maintaining the immunosuppressive role of regulatory T cells in the tumor microenvironment.	[19]
**FRS2**<br>Fibroblast growth factor receptor substrate 2	An intracellular adaptor protein for the FGF receptor, involved in MAPK signaling.<br>Acts as an oncogene in ovarian cancer and is associated with poor prognosis in osteosarcoma patients.	[20]<br>[21]
**RASA1**<br>RasGTPase-activating protein 1 (p21 activator)	A negative regulator of Ras-MAPK and Ras-Akt proliferative signaling by enhancing Ras's GTP-hydrolyzing activity in response to elevated cytosolic calcium ions.<br>Known tumor suppressor gene targeted by the oncogenic miR-21.	[22]<br>[23]

NF-κB, Nuclear Factor Kappa B; FGF, fibroblast growth factor; MAPK, Mitogen-Activated Protein Kinase; Ras-Akt, Ras-Protein Kinase B Signaling Pathway; Ras-MAPK, Ras-Mitogen-Activated Protein Kinase.

REFERENCES:

1. Krapivinsky, G.; Krapivinsky, L.; Stotz, S. C.; Manasian, Y.; Clapham, D. E. POST, Partner of Stromal Interaction Molecule 1 (STIM1), Targets STIM1 to Multiple Transporters. *Proc. Natl. Acad. Sci. U. S. A.* **2011**, *108* (48), 19234–19239. DOI: 10.1073/pnas.1117231108. PMID: 22084111; PMCID: PMC3228472.

2. Kline, S. L.; Cheeseman, I. M.; Hori, T.; Fukagawa, T.; Desai, A. The Human Mis12 Complex Is Required for Kinetochore Assembly and Proper Chromosome Segregation. *J. CellBiol.* **2006**, *173* (1), 9–17. DOI: 10.1083/jcb.200509158. PMID: 16585270; PMCID: PMC2063780.

3. Wang, X.; Lu, X.; Wang, P.; Chen, Q.; Xiong, L.; Tang, M.; Hong, C.; Lin, X.; Shi, K.; Liang, L.; Lin, J. SRSF9 Promotes Colorectal Cancer Progression via Stabilizing DSN1 mRNA in an m6A-Related Manner. *J. Transl. Med.* **2022**, *20* (1), 198. DOI: 10.1186/s12967-022-03399-3. PMID: 35509101; PMCID: PMC9066907.

4. Sun, C.; Huang, S.; Ju, W.; et al. Elevated DSN1 Expression Is Associated with Poor Survival in Patients with Hepatocellular Carcinoma. *Hum. Pathol.* **2018**, *81*, 113–120. https://doi.org/10.1016/j.humpath.2018.06.032.

5. Peng, Q.; Wen, T.; Liu, D.; Wang, S.; Jiang, X.; Zhao, S.; Huang, G. DSN1 Is a Prognostic Biomarker and Correlated with Clinical Characterize in Breast Cancer. *Int. Immunopharmacol.* **2021**, *101* (Pt B), 107605. DOI: 10.1016/j.intimp.2021.107605. PMID: 34238686.

6. Pant, D. C.; Dorboz, I.; Schluter, A.; Fourcade, S.; Launay, N.; Joya, J.; Aguilera-Albesa, S.; Yoldi, M. E.; Casasnovas, C.; Willis, M. J.; Ruiz, M.; Ville, D.; Lesca, G.; Siquier-Pernet,

K.; Desguerre, I.; Yan, H.; Wang, J.; Burmeister, M.; Brady, L.; Tarnopolsky, M.; Cornet, C.; Rubbini, D.; Terriente, J.; James, K. N.; Musaev, D.; Zaki, M. S.; Patterson, M. C.; Lanpher, B. C.; Klee, E. W.; Pinto, E. V.; Vairo, F.; Wohler, E.; Sobreira, N. L. M.; Cohen, J. S.; Maroofian, R.; Galehdari, H.; Mazaheri, N.; Shariati, G.; Colleaux, L.; Rodriguez, D.; Gleeson, J. G.; Pujades, C.; Fatemi, A.; Boespflug-Tanguy, O.; Pujol, A. Loss of the Sphingolipid Desaturase DEGS1 Causes Hypomyelinating Leukodystrophy. *J. Clin. Invest.* **2019**, *129* (3), 1240–1256. DOI: 10.1172/JCI123959.

7. Blackburn, N. B.; Michael, L. F.; Meikle, P. J.; Peralta, J. M.; Mosior, M.; McAhren, S.; Bui, H. H.; Bellinger, M. A.; Giles, C.; Kumar, S.; Leandro, A. C.; Almeida, M.; Weir, J. M.; Mahaney, M. C.; Dyer, T. D.; Almasy, L.; VandeBerg, J. L.; Williams-Blangero, S.; Glahn, D. C.; Duggirala, R.; Kowala, M.; Blangero, J.; Curran, J. E. Rare DEGS1 Variant Significantly Alters De Novo Ceramide Synthesis Pathway. *J. LipidRes.* **2019**, *60* (9), 1630–1639. DOI: 10.1194/jlr.P094433.

8. Zhang, D.; Tomisato, W.; Su, L.; Sun, L.; Choi, J. H.; Zhang, Z.; Wang, K. W.; Zhan, X.; Choi, M.; Li, X.; Tang, M.; Castro-Perez, J. M.; Hildebrand, S.; Murray, A. R.; Moresco, E. M. Y.; Beutler, B. Skin-Specific Regulation of SREBP Processing and Lipid Biosynthesis by Glycerol Kinase 5**.** *Proc. Natl. Acad. Sci. U. S. A.* **2017**, *114* (26), E5197–E5206. DOI: 10.1073/pnas.1705312114.

9. Yan, M.; Wu, Y.; Peng, W.; et al. Exposure to Particulate Matter 2.5 and Cigarette Smoke Induces the Synthesis of Lipid Droplets by Glycerol Kinase 5. *Clin. Exp. Pharmacol. Physiol.* **2021**, *48*, 498–507. DOI: 10.1111/1440-1681.13463.

10. Parker, J. L.; Deme, J. C.; Kolokouris, D.; et al. Molecular Basis for Redox Control by the Human Cystine/Glutamate Antiporter System Xc−. *Nat. Commun.* **2021**, *12*, 7147. DOI: 10.1038/s41467-021-27414-1.

11. Lin, W.; Wang, C.; Liu, G.; Bi, C.; Wang, X.; Zhou, Q.; Jin, H. SLC7A11/xCT in Cancer: Biological Functions and Therapeutic Implications. *Am. J. CancerRes.* **2020**, *10* (10), 3106–3126.

12. Liu, M. R.; Zhu, W. T.; Pei, D. S. System Xc−: A Key Regulatory Target of Ferroptosis in Cancer. *Invest. New Drugs* **2021**, *39*, 1123–1131. DOI: 10.1007/s10637-021-01070-0.

13. Tu, H.; Costa, M. XIAP's Profile in Human Cancer. *Biomolecules* **2020**, *10,* 1493. DOI: 10.3390/biom10111493.

14. Fukazawa, H.; Noguchi, K.; Masumi, A.; Murakami, Y.; Uehara, Y. BimEL Is an Important Determinant for Induction of Anoikis Sensitivity by Mitogen-Activated Protein/Extracellular Signal-Regulated Kinase Kinase Inhibitors. *Mol. Cancer Ther.* **2004**, *3* (10), 1281–1288. PMID: 15486195.

15. Patel, D.; Morton, D. J.; Carey, J.; Havrda, M. C.; Chaudhary, J. Inhibitor of Differentiation 4 (ID4): From Development to Cancer. *Biochim. Biophys. Acta* **2015**, *1855* (1), 92–103. DOI: 10.1016/j.bbcan.2014.12.002.

16. Chen, H. J.; Yu, Y.; Sun, Y. X.; Huang, C. Z.; Li, J. Y.; Liu, F.; Guo, G. X.; Ye, Y. B. Id4 Suppresses the Growth and Invasion of Colorectal Cancer HCT116 Cells through CK18-

Related Inhibition of AKT and EMT Signaling. *J. Oncol.* **2021**, *2021*, 6660486. DOI: 10.1155/2021/6660486.

17. Carey, J. P.; Asirvatham, A. J.; Galm, O.; Ghogomu, T. A.; Chaudhary, J. Inhibitor of Differentiation 4 (Id4) Is a Potential Tumor Suppressor in Prostate Cancer. *BMC Cancer* **2009**, *9*, 173. DOI: 10.1186/1471-2407-9-173.

18. Baker, L. A.; Holliday, H.; Swarbrick, A. ID4 Controls Luminal Lineage Commitment in Normal Mammary Epithelium and Inhibits BRCA1 Function in Basal-Like Breast Cancer. *Endocr.-Relat. Cancer* **2016**, *23* (9), R381–R392. DOI: 10.1530/ERC-16-0196.

19. O'Neill, T. J.; Tofaute, M. J.; Krappmann, D. Function and Targeting of MALT1 Paracaspase in Cancer. *Cancer Treat. Rev.* **2023**, *117*, 102568. DOI: 10.1016/j.ctrv.2023.102568.

20. Luo, L. Y.; Kim, E.; Cheung, H. W.; Weir, B. A.; Dunn, G. P.; Shen, R. R.; Hahn, W. C. The Tyrosine Kinase Adaptor Protein FRS2 Is Oncogenic and Amplified in High-Grade Serous Ovarian Cancer. *Mol. Cancer Res.* **2015**, *13* (3), 502–509. DOI: 10.1158/1541-7786.MCR-14-0407.

21. Zhu, Y.; Liu, Z.; Cao, L.; Fan, G.; Ji, R.; Zhang, L.; Daji, S.; Zhu, H.; Wang, Y.; Zhou, G. FRS2 Regulated by miR-429 and miR-206 Promotes Angiogenesis in Osteosarcoma. *Gene* **2024**, *898*, 148118. DOI: 10.1016/j.gene.2023.148118.

22. Zhang, Y.; Li, Y.; Wang, Q.; Su, B.; Xu, H.; Sun, Y.; Sun, P.; Li, R.; Peng, X.; Cai, J. Role of RASA1 in Cancer: A Review and Update (Review). *Oncol. Rep.* **2020**, *44* (6), 2386–2396. DOI: 10.3892/or.2020.7807.

23. Zhang, L.; Zhan, X.; Yan, D.; Wang, Z. Circulating MicroRNA-21 Is Involved in Lymph Node Metastasis in Cervical Cancer by Targeting RASA1. *Int. J. Gynecol. Cancer* **2016**, *26* (5).
